# Two resected cases of benign adenomyoepithelioma

**DOI:** 10.1186/s40792-023-01793-7

**Published:** 2023-12-20

**Authors:** Yurika Fukudome, Yoshika Nagata, Yui Yamada, Toshihiro Saeki, Takahisa Fujikawa

**Affiliations:** 1 Department of Surgery, Kokura Memorial Hospital, 3-2-1 Asano, Kokurakita-Ku, Kitakyushu City, Fukuoka, 802-8555 Japan; 2 Department of Pathology, Kokura Memorial Hospital, 3-2-1 Asano, Kokurakita-Ku, Kitakyushu City, Fukuoka, 802-8555 Japan

**Keywords:** Adenomyoepithelioma, Breast, Apparent diffusion coefficient, Surgical resection

## Abstract

**Background:**

Adenomyoepithelioma (AME) of the breast is an uncommon tumor characterized by the proliferation of ductal epithelial and myoepithelial cells with the heterogeneity. Although benign AME is relatively easy to differentiate from breast cancer by core needle biopsy (CNB) alone, a definitive diagnosis is often difficult. The imaging findings of AME are also variable, and there are particularly few reports about radiological features, including contrast-enhanced magnetic resonance imaging (MRI) and apparent diffusion coefficient (ADC) values in AME.

**Case presentation:**

We present two cases of benign AME. Case 1 is a 30-year-old woman with a history of asthma. The cystic tumor shows smooth borders, and the intracystic solid component is irregular in shape and high vascularity. The pathological findings of the tumor were benign on CNB. The MRI scan showed a decreased ADC value. Case 2 is a 60-year-old woman with only a history of arrhythmia. The tumor shows a lobulated mass with cystic space and coarse calcifications. The pathological findings of the tumor were found to be benign by CNB. Dynamic MRI scan showed a fast washout pattern with a decreased ADC value. Both patients underwent excisional biopsy to confirm the diagnosis, and the pathological diagnosis was benign AME in both cases.

**Conclusions:**

The AME of the breast has little specific imaging information, so it can be difficult to diagnose based on pathological findings of biopsy specimen. In our case, the ADC values were exceptionally low, contrary to previous reports. It is essential to carefully diagnose AME, considering the discrepancies in imaging findings observed in this case.

## Background

Adenomyoepithelioma (AME) of the breast is a rare benign tumor characterized by the proliferation of ductal epithelial and myoepithelial cells with variable clinical and diagnostic features [1]. Histopathologically, benign AME is relatively easy to differentiate from breast cancer. Due to the variable histological and morphologic spectrum of AME, a core needle biopsy (CNB) alone is frequently insufficient to establish a definitive diagnosis. [2, 3].

The imaging studies, including contrast-enhanced magnetic resonance imaging (MRI), and matching the pathology of the breast tumor with the imaging findings are important for diagnosis. In addition, an apparent diffusion coefficient (ADC) value on MRI is typically helpful in distinguishing between benign and malignant tumors of the breast [4, 5], however there were few reports about ADC value in AME.

We herein report that two cases of benign AME identified by excisional biopsy. As one case involved a cystic mass and the other a growing tumor, malignancy was ruled out through surgical intervention. In these two cases, MRI demonstrated lower ADC values, contrary to the findings of earlier investigations.

## Case presentation

**Fig. 1:**
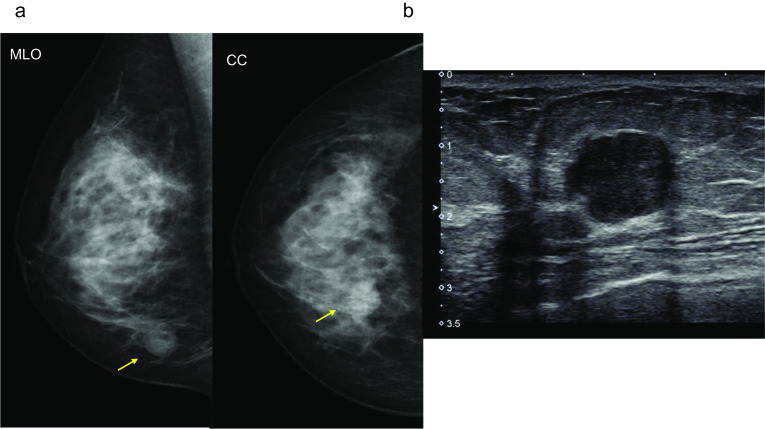
**A** Mammography (MG) shows a high-density mass with circumscribed margins (arrow). **B** Intracystic solid component was irregular shape and high vascularity in ultrasonography (US)

**Fig. 2:**
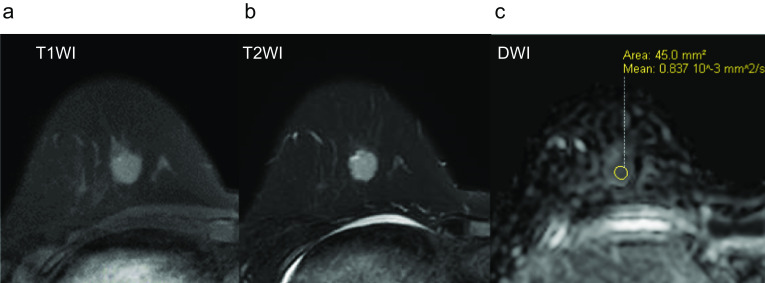
Magnetic resonance imaging (MRI) showed the tumor detected as equal signal intensity (SI) on T1-weighted images (WI) (**A**), and high SI on T2-WI (**B**). **C** This tumor showed mild diffusion restriction on diffusion-weighted imaging (DWI) (ADC value 0.837 × 10–3 mm2/sec)

**Fig. 3:**
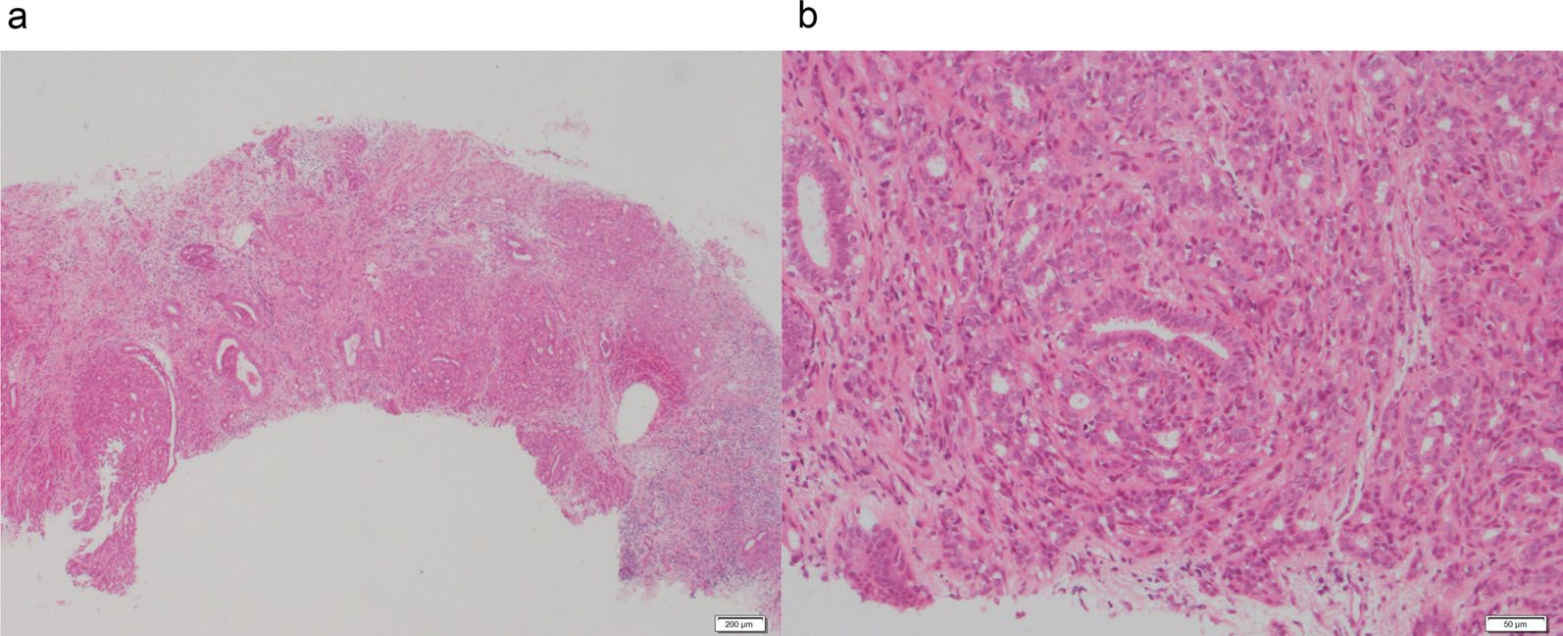
Core needle biopsy (CNB) showed proliferation of ductal epithelial cells without nuclear atypia, diagnosed as intraductal papilloma (**A**, low power view; **B**, high power view)

**Fig. 4:**
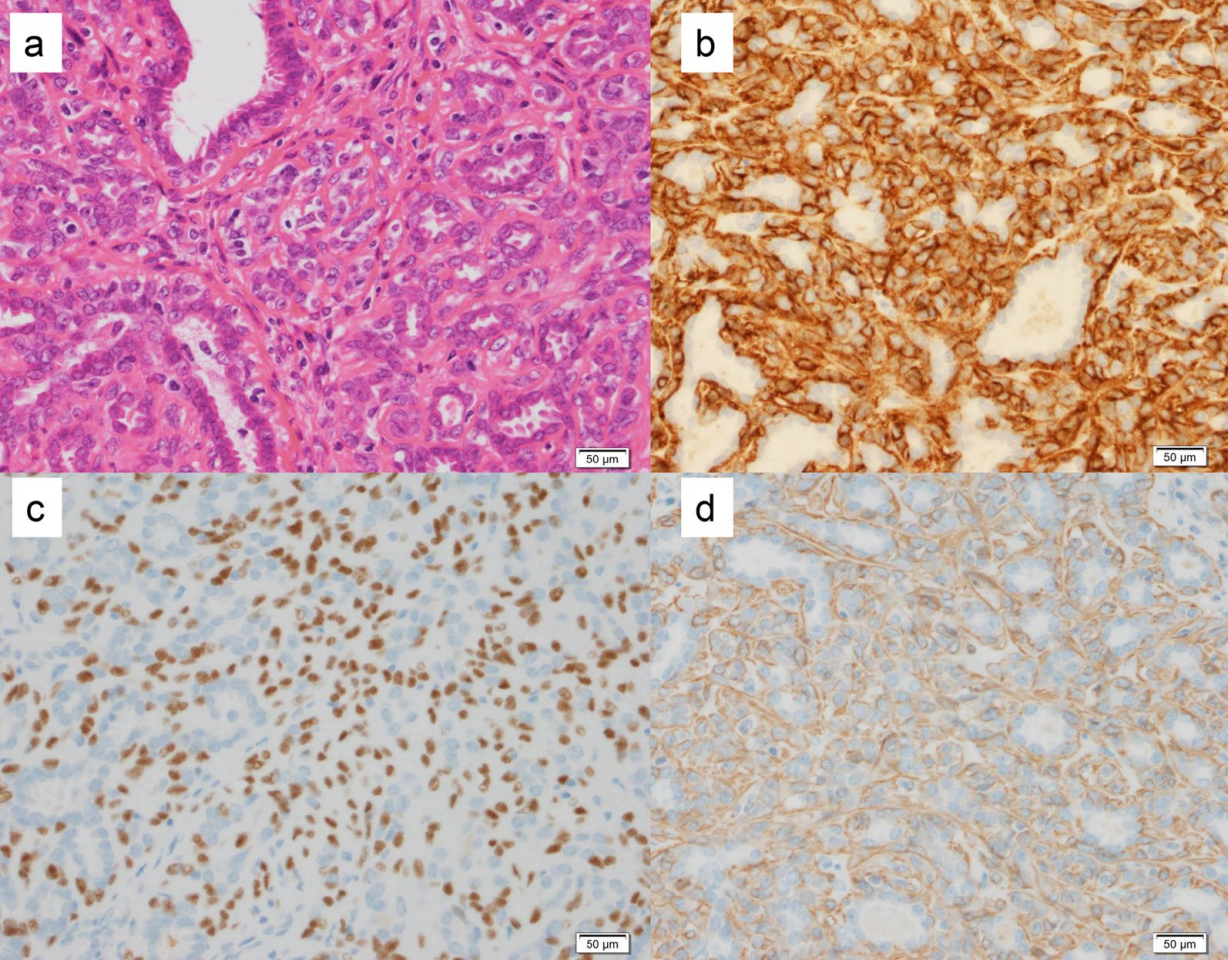
**A** Histologically, the tumor showed biphasic proliferation of ductal epithelial cells and myoepithelial cells. **B**–**D** The myoepithelial cells were positive for Cluster designation 10 (CD10), p63, and alpha smooth muscle actin (SMA) by immunohistological staining

Case 1: A 30-year-old woman show a focal asymmetric density (FAD) in her right breast on a mammography (MG) for breast cancer screening. The patient was tested by US of breast screening 4 years before, there was no abnormal findings at the time. She has a notable medical history of asthma, and there is no noteworthy family history. The physical examination revealed an elastic hard lump with a smooth surface and clear margins located in the inner lower quadrant of the right breast. No skin changes or dimpling were observed. There were no inflammatory signs and no palpable lymphadenopathy. All laboratory data were unremarkable. MG shows a well-defined round shaped mass in the lower and inner regions of the right breast, that could be classified as category 3　(Fig. 1a). Ultrasonography (US) indicated a cystic tumor with smooth borders measuring 1.6 cm in diameter. The tumor was nearly oval in shape with a partially lobulated, a hypoechoic lesion was detected in the cystic wall. The intracystic solid component was irregular shape and high vascularity, and classified as categoly 4 (Fig. 1b). MRI showed the lobulated tumor with well-defined margin detected as same signal intensity (SI) on T1-weighted images (WI), and high SI on T2-WI. This tumor was diffusion restriction on diffusion-weighted imaging (DWI) (ADC value 0.837 × 10^–3^ mm^2^/sec) (Fig. 2). CNB shows that glandular ducts are mostly made up of ductal epithelial cells without nuclear atypia. The stroma has lymphocytic infiltration and weak fibrosis. There were no obvious malignant findings, and the tumor was diagnosed as benign tumor such as fibroadenoma (FA) or intraductal papilloma (Fig. 3a, b). Since the intracystic carcinoma could not be completely ruled out, an excisional biopsy was performed. Grossly, the tumor was a white nodular lesion with relatively clear borders. Histologically, the tumor showed lobular growth with biphasic proliferation of ductal epithelial cells and myoepithelial cells (Fig. 4a). The duct dilatation forming grouped cysts, and lymphocytic infiltration were seen in some part of the tumor. The myoepithelial cells were positive for Cluster designation 10 (CD10), p63, and alpha smooth muscle actin (SMA) by immunohistological staining (Fig. 4b–d). There were no malignant findings in either the ductal epithelial cells or the myoepithelial cells. Based on these features, the pathological diagnosis was the AME. The surgical margins were negative. The patient has no evidence of recurrence after surgery.

**Fig. 5:**
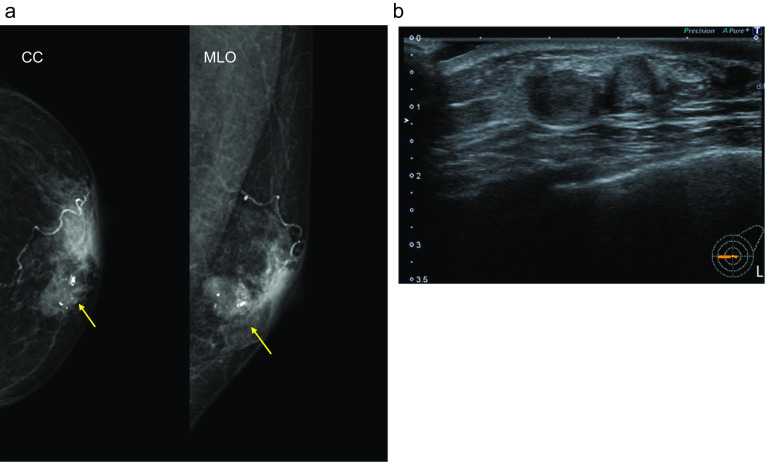
**A** MG shows a well-circumscribed mass with the classic, coarse calcifications. **B** US indicated a dumbbell-shaped hypoechoic tumor

**Fig. 6:**
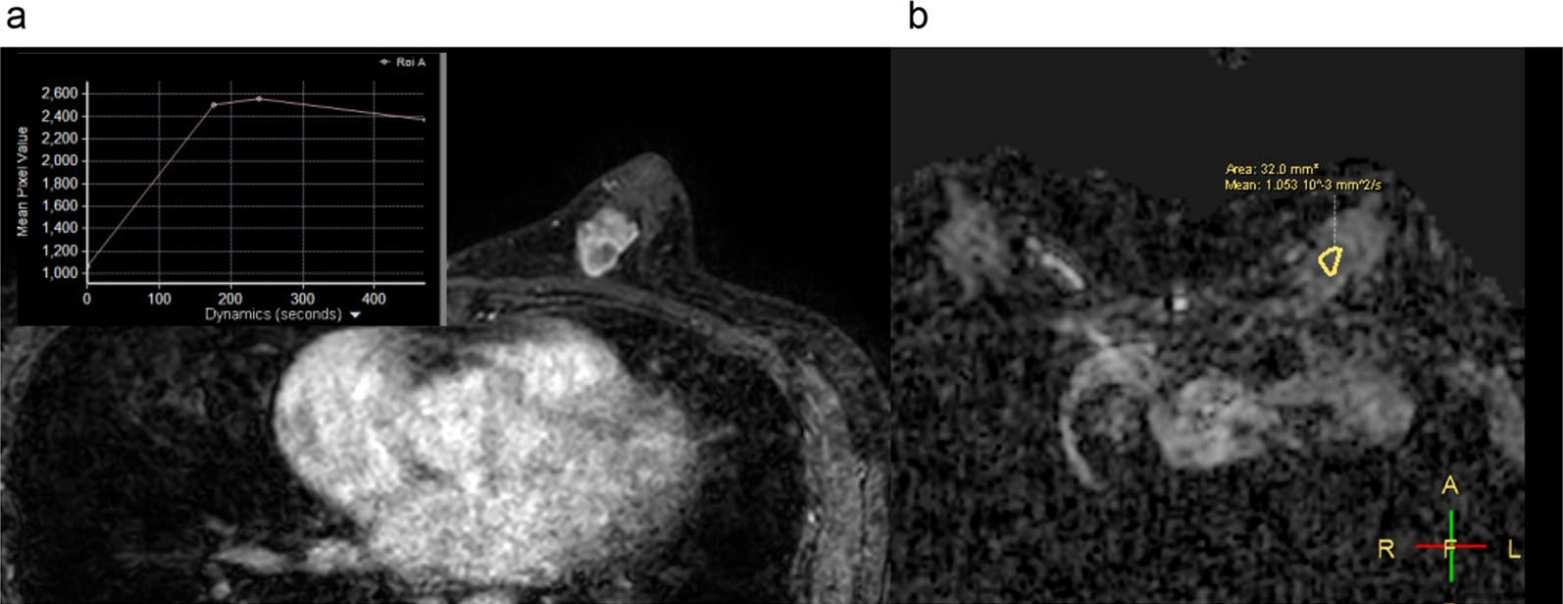
**A **MRI shows the irregular shaped mass enhanced with fast-washout kinetic pattern. **B** The tumor shows high signal of DWI with high diffusion restriction (ADC value 1.053 × 10^–3^ mm^2^/sec)

Case 2: A 60-year-old woman showed a tumor in her left breast on a MG for breast cancer screening 7 years ago. The tumor size was 1.5 cm in diameter, and CNB revealed a benign tumor such as adenosis or ductal adenoma. A MG taken for breast cancer screening showed the growing mass 7 years later, prompting her to revisit our hospital. Her medical history was arrhythmia treated with ablation, and there was no remarkable family history. The physical examination revealed an elastic hard lump with dimpling sign in the inner lower quadrant of the left breast. All laboratory data were unremarkable. 3D-MG (tomosynthesis) shows a well-circumscribed lobulated isodense mass with the coarse calcifications and some amorphous calcification (Fig. 5a). These calcifications have not changed compared to 7 years ago. US indicated a dumbbell-shaped hypoechoic, and hyper vascularity tumor measuring 2.2 cm in diameter (Fig. 5b). MRI showed the irregular shaped mass with heterogeneous enhancement. The internal contrast poor zone shows high signal on T1WI, T2WI and DWI with high diffusion restriction (ADC value 1.053 × 10–3 mm2/sec), and considered to be necrotic. The time-signal intensity curve of the tumor shows a fast washout pattern (Fig. 6). The non-mass like enhancements (NMEs) suspected intraductal extension to the nipple. There was no obvious tumor invasion into skin or pectoralis major muscle.

**Fig. 7:**
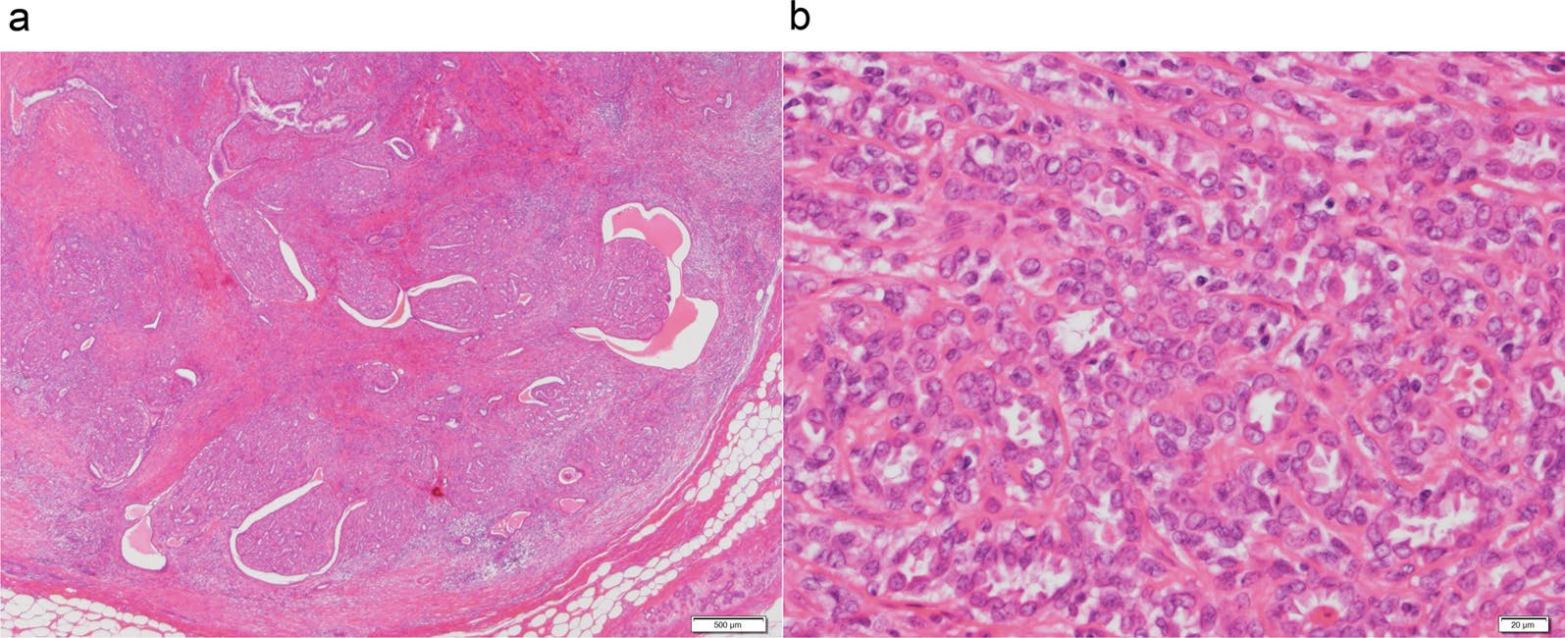
The vacuum-assisted breast biopsy (VAB) shows the biphasic proliferation of both glandular and myoepithelial cells, and the tumor was diagnosed as a benign tumor such as ductal adenoma or intraductal papilloma (**A**, low power view; **B**, high power view)

**Fig. 8:**
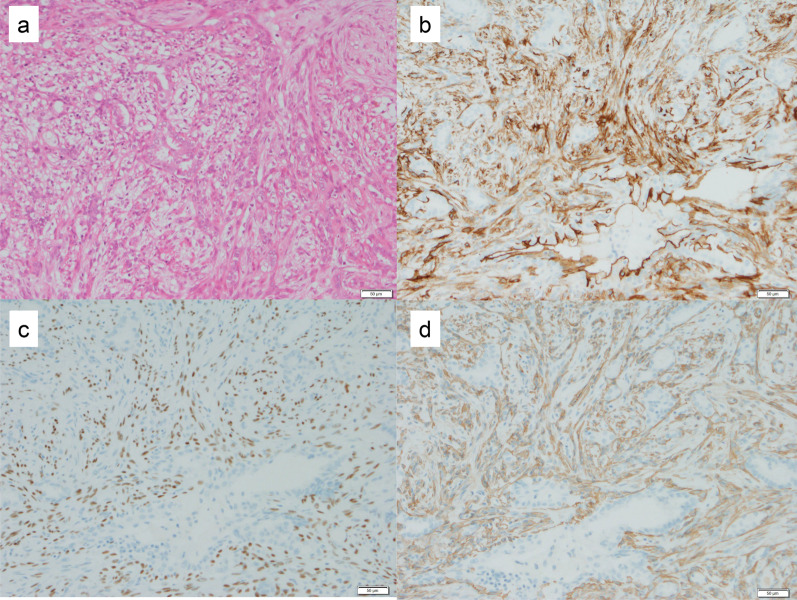
**A** Histologically, the tumor showed the biphasic proliferation of epithelial and myoepithelial cells. Some myoepithelial cells show the spindle shaped with fascicular pattern. **B**–**D** Immunohistological labeling revealed that the myoepithelial cells exhibited positivity for CD10, p63, and alpha SMA

**Fig. 9:**
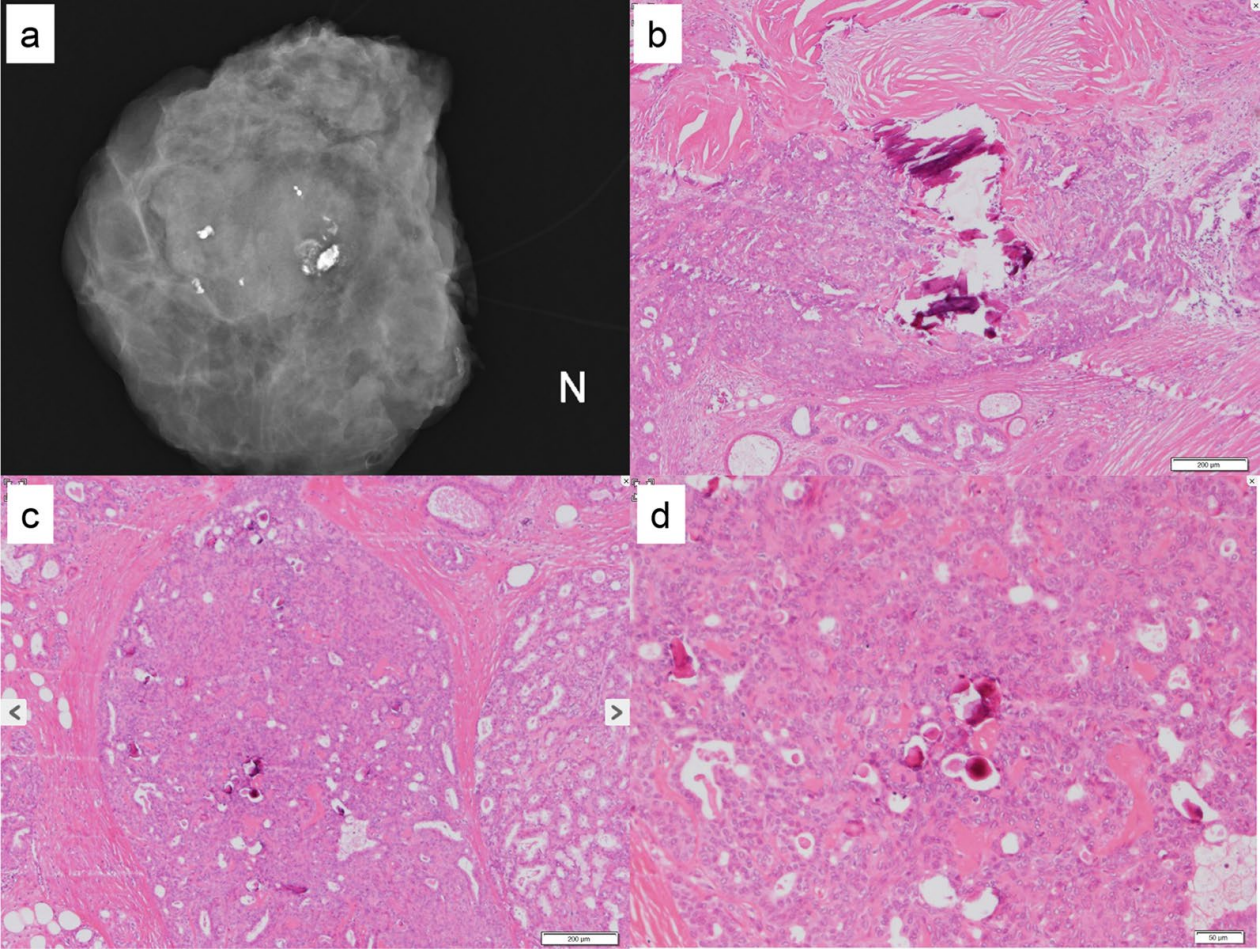
**A** MG imaging of the extracted specimen revealed coarse calcifications concentrated within the tumor. **B** Histologically, hyalinization and fibrosis were present around the coarse calcifications that were identified in the stromal tissue. **C**, **D** Amorphous and punctate calcifications were confirmed within the mammary ductal epithelium by H.E. staining

The vacuum-assisted breast biopsy (VAB) shows the biphasic proliferation and dedifferentiation of both glandular and myoepithelial cells. The epithelial components also show apocrine differentiation. There were no obvious malignant findings, and the tumor was diagnosed as benign tumor such as ductal adenoma or intraductal papilloma (Fig. 7). These pathological findings were almost similar to those of a previous CNB. The tumor had gradually increased over the past 7 years, and surgery was performed to exclude malignancy. Grossly, the tumor was a white, well-defined nodular lesion. Histologically, the tumor showed the biphasic proliferation of ductal epithelial and myoepithelial cells (Fig. 8a). Some myoepithelial cells show the spindle shaped with bundle-like proliferation. Immunohistological labeling revealed that the myoepithelial cells exhibited positivity for CD10, p63, and alpha SMA (Fig. 8b–d). In the stromal tissue, there were coarse calcifications along with surrounding hyalinization and fibrosis. The cyst within the tumor ruptured and was accompanied by foamy cells and histiocytes, which seem to have undergone hyalinization and fibrosis over time. While the morphology of the calcifications resembled that typically associated with old FA, the structure of the background mammary gland differed from that of FA. MG imaging of the extracted specimen revealed coarse calcifications localized within the tumor, along with amorphous and punctate calcifications in the surrounding mammary gland tissue (Fig. 9a, b). Amorphous and punctate calcifications were confirmed within the mammary ductal epithelium, leading to the diagnosis of benign secretory calcification (Fig. 9c, d). There were no malignant findings in either the ductal epithelial cells or the myoepithelial cells. Based on these features, the pathological diagnosis was the benign AME. The resected margins were negative, and the patient has no evidence of recurrence after surgery.

## Discussion

AME, first described by Hamperl in 1970, is an epithelial tumor in which both glandular epithelial cells and myoepithelial cells of the mammary gland show proliferation [1]. AME is a rare tumor of the mammary gland, and a few cases of local recurrence, distant metastasis, and death with varying biological characteristics have been reported.

The median size of AME is approximately 1.5–2.5 cm [2, 6, 7]. The clinical presentation is often characterized by a single breast nodule forming a well-defined mass lesion. The round or lobulated masses with well-defined or partially indistinct borders were seen on MG and US [3]. The tumor had a partially obscured margin, and cystic changes or necrosis may be present. Malignant AMEs tend to have indistinct margins, marked architectural distortion on MG. Tumors accompanied by calcifications on MG are exceptionally rare in AME, comprising less than 5% of reported cases to date [8]. Microcalcifications with blurred borders and internally grouped macrocalcifications were documented as a case report of AME. In our case, coarse calcifications are localized in the stromal tissue of AME tumors, while amorphous and punctate calcifications are commonly found in both tumors and mammary glands. We evaluated the presence of coarse calcifications associated with AME.

At breast MRI, AME usually present as low to isointense on T1WI and hyperintense mass on T2WI [8]. The imaging findings on T2WI are similar to those of phyllodes tumors and mixed type mucinous carcinomas. AME shows heterogeneous enhancement with washout or plateau enhancement kinetics in a dynamic study. A washout enhancement pattern tends to show malignant AME [8, 9].

The mean ADC of malignant tumors was approximately 0.80–1.03 × 10^–3^ mm^2^/sec, which was significantly lower than that of benign lesions [4, 5]. Therefore, the ADC threshold of 1.00 × 10^–3^ mm^2^/sec can be recommended for distinguishing breast cancers from benign lesions [4]. According to a recently released prospective multicenter study, a cutoff value for the ADC of 1.53 × 10^–3^ mm^2^/sec can reduce the biopsy rate by 20.9% without lowering sensitivity [10]. While there is a wealth of information regarding the measurement of ADC in breast cancer, the limited number of cases has resulted in a scarcity of publications on ADC in AME. Table 1 provides an overview of ADC values in benign and malignant mammary tumors [4, 5, 11], as well as in AME and breast cancer. In our case, the ADC values were 0.837 and 1.053 mm2/s, respectively. To the best of our knowledge, there have been no reports of benign AME with such a remarkably low ADC value. A benign tumor with high cellularity, as seen in cases of papillary lesions, ductal ectasia, cystic components in our study, may demonstrate a low ADC. Furthermore, lymphocyte infiltration surrounds the tumor, especially in Case 1, which could be expected to decrease ADC.

**Table 1:** ADC values in benign and malignant breast tumors and AME

Histopathological type	Author	Publication year	T1 WI (SI)	T2 WI (SI)	ADC value (10^–3^ mm^2^/sec)	
Benign AME	Zhang L et al.[9]	2016	Iso	High	1.54	
	Zhang L et al.[9]	2016	Low	High	1.61	
	Zhang L et al.[9]	2016	Low	High	1.69	
	Takenaka J et al.*	2020	High	High	1.264	
	Nakaguchi K et al.*	2008	High	High	1.2	
	Present case	2023	Iso	High	0.837	
	Present case	2023	High	High	1.053	
					0.837–1.69 (Average 1.31, Mean 1.264)	
Malignant AME						
	Zhang L et al.[9]	2016	Slight low	Slight high	1.15	
Benign Breast tumor						
	Surov A et al.[4]	2019	ND **	ND	1.5	
	Bickel H et al.[11]	2023	ND	ND	1.45	
Malignant Breast tumor (Breast cancer)						
	Surov A et al.[4]	2019	ND	ND	1.03	
	Bickel H et al.[11]	2023	ND	ND	0.95	

*Citation not available; **ND: not described

## Conclusions

In our case, breast tumors show decreased ADC values on breast MRI. It is essential to carefully diagnose AME, considering the discrepancies in imaging findings observed in this case. In order to understand the characteristics of AME and make an accurate diagnosis, it is important to accumulate more cases and conduct further evaluation.

## Data Availability

All the data and materials used in this study were obtained from publicly available sources or databases, and all cited literature is accessible through PubMed.

## Abbreviations

AME: Adenomyoepithelioma
ADC: Apparent diffusion coefficient
CD: Cluster designation
CNB: Core needle biopsy
DWI: Diffusion-weighted imaging
FAD: Focal asymmetric density
FA: Fibroadenoma
MG: Mammography
MRI: Magnetic resonance imaging
NMEs: Non-mass like enhancements
SI: Signal intensity
SMA: Smooth muscle actin
US: Ultrasonography
VAB: Vacuum-assisted breast biopsy
WI: Weighted images
